# Cholesterol-Depletion-Induced Membrane Repair Carries a Raft Conformer of P-Glycoprotein to the Cell Surface, Indicating Enhanced Cholesterol Trafficking in MDR Cells, Which Makes Them Resistant to Cholesterol Modifications

**DOI:** 10.3390/ijms241512335

**Published:** 2023-08-02

**Authors:** Zsuzsanna Gutay-Tóth, Gabriella Gellen, Minh Doan, James F. Eliason, János Vincze, Lajos Szente, Ferenc Fenyvesi, Katalin Goda, Miklós Vecsernyés, Gábor Szabó, Zsolt Bacso

**Affiliations:** 1 Department of Biophysics and Cell Biology, Faculty of Medicine, University of Debrecen, 4032 Debrecen, Hungary; gutaynetzs@gmail.com (Z.G.-T.); gabgellen@staff.elte.hu (G.G.); vivavn@gmail.com (M.D.); goda@med.unideb.hu (K.G.); szabog@med.unideb.hu (G.S.); 2 Doctoral School of Molecular Cell and Immune Biology, University of Debrecen, 4032 Debrecen, Hungary; 3 MTA-ELTE Lendület Ion Mobility Mass Spectrometry Research Group, Department of Analytical Chemistry, Institute of Chemistry, ELTE Eötvös Loránd University, 1053 Budapest, Hungary; 4 Great Lakes Stem Cell Innovation Center, Detroit, MI 48202, USA; jeliason@gmail.com; 5 Department of Physiology, Faculty of Medicine, University of Debrecen, 4032 Debrecen, Hungary; vincze.janos@med.unideb.hu; 6 CycloLab Cyclodextrin Research & Development Laboratory, Ltd., 1097 Budapest, Hungary; szente@cyclolab.hu; 7 Department of Pharmaceutical Technology, Faculty of Pharmacy, University of Debrecen, 4032 Debrecen, Hungary; fenyvesi.ferenc@pharm.unideb.hu (F.F.); vecsernyes.miklos@pharm.unideb.hu (M.V.)

**Keywords:** ABCB1 transporter, cyclodextrin, membrane repair, raft, trafficking, UIC2

## Abstract

The human P-glycoprotein (P-gp), a transporter responsible for multidrug resistance, is present in the plasma membrane’s raft and non-raft domains. One specific conformation of P-gp that binds to the monoclonal antibody UIC2 is primarily associated with raft domains and displays heightened internalization in cells overexpressing P-gp, such as in NIH-3T3 MDR1 cells. Our primary objective was to investigate whether the trafficking of this particular P-gp conformer is dependent on cholesterol levels. Surprisingly, depleting cholesterol using cyclodextrin resulted in an unexpected increase in the proportion of raft-associated P-gp within the cell membrane, as determined by UIC2-reactive P-gp. This increase appears to be a compensatory response to cholesterol loss from the plasma membrane, whereby cholesterol-rich raft micro-domains are delivered to the cell surface through an augmented exocytosis process. Furthermore, this exocytotic event is found to be part of a complex trafficking mechanism involving lysosomal exocytosis, which contributes to membrane repair after cholesterol reduction induced by cyclodextrin treatment. Notably, cells overexpressing P-gp demonstrated higher total cellular cholesterol levels, an increased abundance of stable lysosomes, and more effective membrane repair following cholesterol modifications. These modifications encompassed exocytotic events that involved the transport of P-gp-carrying rafts. Importantly, the enhanced membrane repair capability resulted in a durable phenotype for MDR1 expressing cells, as evidenced by significantly improved viabilities of multidrug-resistant Pgp-overexpressing immortal NIH-3T3 MDR1 and MDCK-MDR1 cells compared to their parents when subjected to cholesterol alterations.

## 1. Introduction

Recent advancements in methodology have facilitated the measurement of the plasma membrane (PM) cholesterol and sphingomyelin complexes through novel techniques [1,2,3]. These measurements have significant implications for understanding the distribution of PM cholesterol. It has been observed that the outer leaflet of cellular membranes generally contains higher cholesterol levels than the inner leaflet, except in the case of enucleated human red blood cells [4,5]. However, the scientific community has yet to reach a consensus regarding the vertical distribution of PM cholesterol. The quick flip of cholesterol across the leaflets of cellular membranes, which is incongruent with the slow rate of transporters (ABCA1, ABCG1) that influence sterol distribution [6,7], poses a challenge. The transporters cannot match the speed at which cholesterol undergoes trans leaflet flip. These structural insights have significant implications for interpreting cell biological and immunological processes, including cholesterol homeostasis, Wnt or Hedgehog signaling [8,9,10], and the inflammatory or anti-inflammatory functions of macrophages and dendritic cells [11,12]. It is plausible that different cell types exhibit substantial variations in cholesterol distribution. Our research findings are consistent with the stated processes and provide additional insights from a different perspective. Here, the trafficking of a cell surface transporter sensitive to its cholesterol microenvironment was employed to monitor PM cholesterol levels. Our results support the existence of variations in PM cholesterol among immortal cells and illustrate an example of cholesterol redistribution through vesicular transport.

P-glycoprotein (P-gp, MDR1, ABCB1) first caught the attention of scientists because of its involvement in the multidrug resistance (MDR) phenotype of cancer cells. The protein belongs to the ATP-binding cassette (ABC) transporter superfamily and pumps a wide range of lipophilic or amphiphilic compounds in an ATP-dependent way. It is widely observed to be overexpressed in cancer cells and is still considered a substantial factor in most clinical cases of multidrug resistance [13,14,15]; however, it is now clear that the MDR phenotype always involves other factors in addition to P-gp [16,17].

It was an early observation that MDR is also accompanied by the altered lipid composition of the plasma membrane (PM), mainly manifested as the upregulation of lipids and proteins that constitute lipid rafts and caveolar domains, such as elevated levels of cholesterol [18,19,20], sphingolipids [21], glucosylceramide [22,23,24], caveolin-1 and cholesterol-rich caveolae [25,26,27,28,29,30,31]. These results suggest that lipid rafts are increased in several P-gp-overexpressing MDR cell lines, accompanied by upregulation of raft-related cellular processes such as membrane and protein turnover, cholesterol trafficking, and altered cell signaling [32,33]. However, care must be taken not to draw a causal relationship between P-gp expression and the lipid content of rafts because P-gp-overexpressing cell lines, even when transfected with P-gp, are often maintained on chemotherapeutic drugs that may result in additional modifications of the cell membranes [34,35,36,37].

It has been suggested that P-gp contributes to stabilizing membrane microdomains because of its lipid translocation function [38,39,40,41,42,43,44,45,46]. However, its cholesterol transporter function looks unlikely [5,47]. At the same time, P-gp is present in both non-raft and raft domains [25,48,49,50] and is, therefore, able to contribute to the ever-changing lipid content of rafts by “The Induced-Fit Model of Raft Heterogeneity” [51] and with its structural cholesterol [52]. Cholesterol perturbation of the membrane has a crucial impact on the function [42,45,50,52,53,54,55,56,57,58,59] and the trafficking of P-gp [50,59].

The UIC2 mAb, often called a conformation-sensitive antibody, recognizes the transporter in the inward-facing conformation of its catalytic cycle when its composite epitope is displayed on the cell surface [60,61]. The total number of cell surface P-gp molecules can be visualized by selected extracellularly binding mAbs (4E3; MM12.10; 15D3). The UIC2 reactive conformer of P-gp is detergent resistant (TritonX-100; Brij98; CHAPS), indicating that it is raft-associated and exhibits intensive internalization involving caveolae and the actin microfilament system [15,50,62,63,64].

The primary aim of the present study was to assess the trafficking of the raft-conformer P-gp in response to perturbations of cholesterol levels in cells. Cholesterol modifications were carried out with the help of β-cyclodextrins [65].

Our results indicated that cholesterol primarily affected the cell surface content of the UIC2-labeled conformer, resulting in an unexpected increase in this mainly raft-associated fraction in the cell membrane after cholesterol depletion. It was found that trafficking was responsible for cell surface changes in P-gp. The increased exocytosis of the UIC2-reactive conformer indicated compensatory cholesterol export to the cell surface via the delivery of cholesterol-rich raft micro-domains. Moreover, based on some publications [66,67], we hypothesized that the increased exocytosis was part of a universal process of cells: membrane repair. In this process, lysosomes and endosomal vesicles are transported to the cell surface to remove lesions and replenish damaged membrane parts [68] also occurring after cholesterol extraction. The amount of lysosomal exocytosis has been measured after cholesterol depletion of the PM, and it was found to be more significant in the 3T3-MDR1 cell line than in the drug-sensitive parental line. The enhanced lysosome-based membrane repair in drug-resistant 3T3-MDR1 cells suggested a more resistant phenotype against cholesterol alterations. Therefore, the viability of P-gp-expressing and non-expressing cells after cyclodextrin treatments were compared, and it was found that P-gp non-expressing cells were more sensitive to cholesterol modulations of the PM. The hereafter confirmed more stable lysosomes in 3T3-MDR1 cells, even after cholesterol extraction of the PM side by side with the expanded endo-lysosomal compartment and enhanced membrane turnover of MDR cells [16,69,70], was found to be responsible for viability differences of the two cell lines. We think that P-gp expression, perhaps together with other tools of the MDR phenomenon, helps to evolve fitter cancer cells by bringing about a durable phenotype with a more efficient membrane repair process in addition to operating as a multidrug transporter.

## 2. Results

### 2.1. Cholesterol Extraction Increased the Amount of Raft-Associated, UIC2-Binding Conformer of P-gp in Cell Surface Membranes

**Figure 1:**
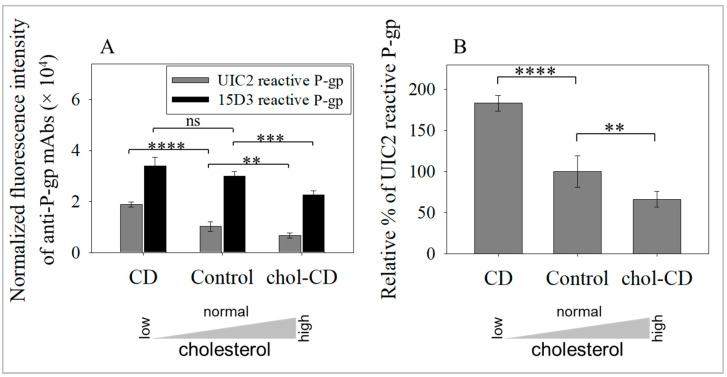
Cell surface levels of the raft-associated UIC2 binding conformer of P-gp increased after cholesterol extraction. (**A**) Effects of modifying cholesterol levels on the binding of the UIC2 and 15D3 mAbs expressed as normalized fluorescence intensities in 3T3-MDR1 cells. Bars represent the mean ± SD values of three-five independent experiments. (**** *p* < 0.0001, *** *p* = 0.0069, ** *p* = 0.01, by unpaired, one-tail *t*-test, ns: non-significant). (**B**) Relative cell surface levels of UIC2 reactive P-gp as a percentage of control samples. Bars represent the mean ± SD values of three-five independent experiments. (**** *p* < 0.0001 and ** *p* = 0.01 by unpaired, one-tail *t*-test.).

We asked whether alterations in the PM’s cholesterol levels affect the cell surface level of different P-gp conformers. We have determined the fluorescence of the total antigen binding capacities (B_max_) of anti-P-gp mAbs, applying them at various concentrations in the presence of cyclodextrin (CD) treatments; CD was used to extract cholesterol from the cell membrane [71], and chol-CD to increase cholesterol levels [72]. An increase in cell-surface P-gp was observed, detected using either the UIC2 or the 15D3 mAbs after cholesterol depletion (Figure 1A). Furthermore, when cholesterol levels were increased, cell surface levels of P-gp transporters were decreased, demonstrating the cholesterol dependence of the phenomenon. To confirm that elevations in antibody binding did not originate from affinity changes, dissociation constants (K_d_) of anti-P-gp mAbs were also determined (Figure S1; supplementary figures are located at the end of this document). Cholesterol level alterations did not significantly modify the affinity of the UIC2 mAb (Figure S1A). However, the affinity of the 15D3 mAb was significantly decreased after cholesterol depletion. When cholesterol levels increased, the change in Kd of the 15D3 mAb was insignificant (Figure S1B). In summary, affinity alterations were either insignificant or tended to fall when cholesterol levels were reduced. Therefore, the measured elevation in antibody binding cannot originate from changes in binding affinity. These data indicated that changes in the expression of P-gp conformers on the PM were inversely proportional to cholesterol levels (Figure 1A). In addition, it must be highlighted that these changes in protein levels affected the raft-associated UIC2 reactive conformer (Figure 1B), which also manifested in total cell surface P-gp level alterations in the 15D3 reactive conformers (Figure 1A).

### 2.2. Cholesterol Depletion-Induced Rise in the UIC2-Binding P-gp Conformer Is the Result of Increased Exocytosis and Not Decreased Endocytosis

It was demonstrated previously in our laboratory that raft-associated UIC2-binding P-gp was internalized significantly faster than the non-raft-associated P-gp molecules [15,50,62,63]. Thus, we hypothesized that the increased cell surface amounts of UIC2-binding P-gp may result from cholesterol-dependent trafficking. Explicitly, it could result from either decreased endocytosis or increased exocytosis. At normal cholesterol, we confirmed the rapid internalization in kinetic measurements, as about half (54%) of the UIC2-binding transporters were found to be internalized after the 20 min incubation (half-life time is 18.5 min).

**Figure 2:**
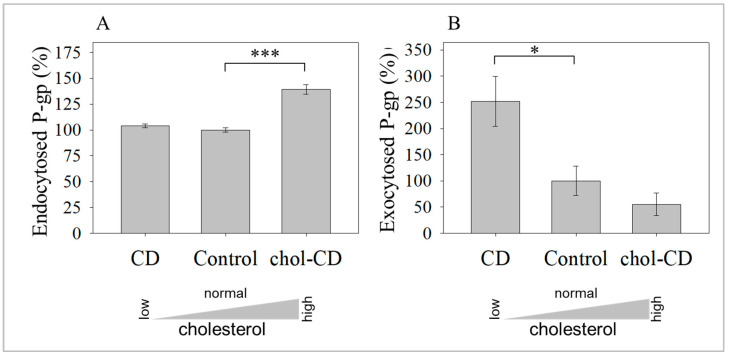
Effect of cholesterol modulations on trafficking of UIC2 reactive P-gp. Effect of treatment with CD or chol-CD on endocytosis (**A**) and exocytosis (**B**) of UIC2 binding P-gp 15 min after treatment of 3T3-MDR1 cells. The results are expressed as a percentage of control. Bars represent the mean ± SD values of three independent experiments. (*** *p* = 0.0001 and * *p* = 0.04 by unpaired, one-tail *t*-test).

This rapid internalization was further enhanced when cholesterol level was increased by chol-CD. The amount of UIC2-binding P-gp endocytosed was 39 ± 5% higher than the control 15 min after the chol-CD treatment (Figure 2A). Surprisingly, cholesterol depletion did not change the internalized fraction of UIC2-binding P-gp significantly: 103 ± 2% compared to the 100 ± 2% control sample. Similar observations were made for total P-gp changes applying the 15D3 mAb [59]. Thus, altered internalization could not explain the increased cell surface amount of UIC2 reactive P-gp. We also examined the kinetics of exocytosis, which behaved oppositely. The rate of exocytosis was increased when the cell membrane was depleted in cholesterol and was reduced after the PM was cholesterol-enriched, as the amount of UIC2-labeled transporters was increased to 250 ± 48% for the CD-treated sample and decreased to 55 ± 21% for the chol-CD treated sample compared to the untreated control (100 ± 28%, Figure 2B), 15 min after cholesterol modulation. These data indicate that alterations in the cholesterol content of the PM extensively modified the exocytosis of the raft-associated fraction of this transmembrane protein.

### 2.3. Inhibition of Exocytosis Prevents the Increase in UIC2-Bound P-gp Caused by Cholesterol Depletion

**Figure 3:**
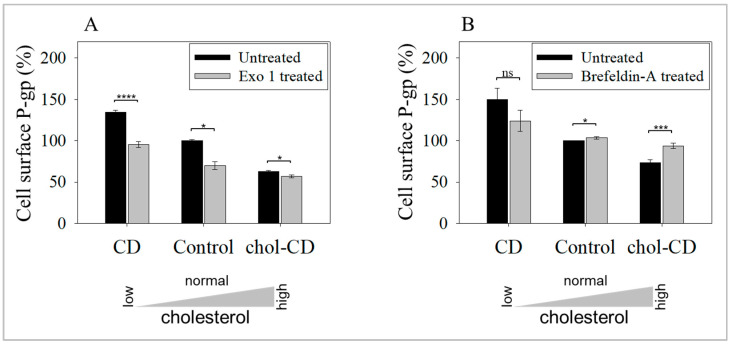
Effect of inhibition of exocytosis on cell surface levels of UIC2 binding P-gp. (**A**) Exo1 decreased cell surface P-gp in all cases (**** *p* < 0.0001 and * *p* = 0.01 by unpaired, one-tail *t*-test). (**B**) BFA only slightly decreased cell surface P-gp in CD-treated samples. Bars represent mean ± SEM values of three independent experiments expressed as a percent of control. (*** *p* = 0.001, * *p* = 0.0379 by unpaired, one-tail *t*-test, ns: non-significant).

To confirm that exocytosis controls the CD-induced increase in UIC2-binding P-gp conformers, we examined the cholesterol-modulated effects in the presence of drugs that inhibit exocytosis. Exo1 decreased the cell surface level of UIC2-bound P-gp in every case (Figure 3A). Cell surface levels were decreased by 38 ± 6% for CD-treated low cholesterol cells, 29 ± 7% for control cells, and 6 ± 5% for the chol-CD treated high cholesterol cells. The inhibition pattern is compatible with the concept of a significant role for exocytosis in this process. The drug also decreased cell surface levels of UIC2-binding P-gp on control cells (Figure 3A, middle bars), indicating the intensive trafficking of these conformers, involving both endo- and exocytosis. The low degree of inhibition in the chol-CD-treated cells probably reflects that the increased cholesterol level already inhibits exocytosis. The most significant inhibition was observed with the CD-treated cells. As expected, the addition of Exo 1 to CD-treated cells returned cell surface levels to those of the control cells.

The effect of brefeldin-A (BFA) was less pronounced with cholesterol-depleted cells (Figure 3B), and in the case of cholesterol supplementation or at undisturbed cholesterol contents, the cell surface level of the UIC2 reactive conformer was increased. The inhibitor, Exo1, is thought to inhibit ARF1 function in an ARF-GAP-dependent manner in the Golgi [73]. Morphologically, Exo1 induces rapid redistribution of the Golgi content back to the ER by transforming the protein and lipid biosynthetic vesicular system into a tubular form [74,75]. The second inhibitor, BFA, is a noncompetitive inhibitor of the ARF1-GDP/GTP exchange factor and therefore inhibits many downstream cytosolic effectors of ARF1 [74,76]. These effectors include the assembly of coat-protein complexes [73], where COPI localized on Golgi, GGA on the TGN and ERC, and adaptor protein complexes AP1 and AP3-involved in protein delivery to lysosomes. With the inhibition of these complexes, BFA disrupts Golgi, but unlike Exo1, it affects vesicular transport at all secretory, endocytic, and recycling pathways. BFA decreased the cell surface level of UIC2-binding conformers only in the CD-treated samples with the highest degree of cell surface-directed traffic of the P-gp. The disturbance of both trafficking systems may have led to unaltered levels in the BFA-only treated control samples, confirming a strict balance in this conformer’s endo- and exocytosis. In the chol-CD treated cell membranes, the increase in P-gp level was likely the result of altered endocytosis, as exocytosis was already downregulated.

### 2.4. UIC2-Binding P-gp Represents the Cholesterol-Dependent Trafficking of Rafts

Perturbation of PM cholesterol levels initiates several compensatory mechanisms [74,77,78,79,80]. Cholesterol saturation leads to an increased redistribution of cholesterol into intracellular pools, and this cholesterol overload causes a reduction in cholesterol accumulation until the PM cholesterol returns to its steady-state level. In contrast, cholesterol depletion induces processes that reestablish the missing PM cholesterol. Mechanisms include induction of cholesterol synthesis in the ER and delivery of cholesterol to the PM via vesicular and non-vesicular pathways, such as trafficking of raft structures through the secretory pathway and movement by cytosolic cholesterol transporter proteins, or perhaps by ER contact sites [81]. Cholesterol depletion of the PM resulted in increased exocytosis of the raft-associated UIC2 binding conformer of P-gp (Figure 2B), which was possible to block with drugs, affecting the secretory pathway (Figure 3). Cholesterol overload, however, blocked further delivery of the protein to the PM. The kinetics of the exocytotic events were fast; they occurred on a time scale of 15–30 min. From these observations, we conclude that the UIC2 binding conformers showed trafficking with raft domains to compensate for cholesterol perturbations.

### 2.5. Exocytosis of LAMP1 and LAMP2 Was Enhanced after Cyclodextrin Treatment in a Calcium and Cholesterol-Dependent Manner

**Figure 4:**
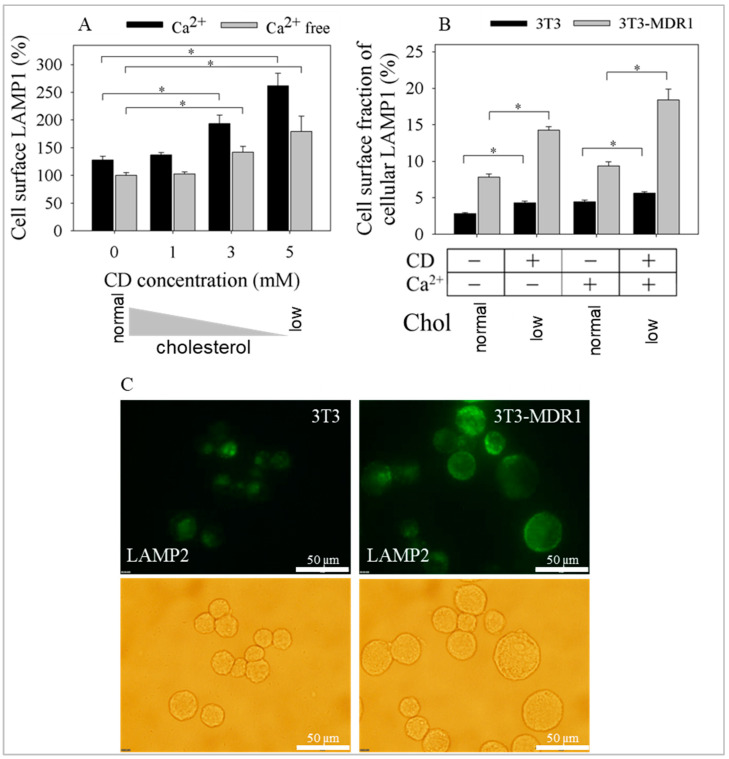
Effect of cholesterol depletion on lysosomal exocytosis. Effect of cholesterol depletion and extracellular calcium on the expression of LAMP1 and LAMP2 proteins on NIH-3T3 and 3T3-MDR1 cell surfaces. (**A**) LAMP1 increased concentration dependent on CD treatment in 3T3-MDR1 cells in the presence or absence of EC Ca^2+^. Bars represent the mean ± SD of three experiments expressed as the percentage of the CD-untreated Ca^2+^-free control sample. A one-way ANOVA was performed with Tukey–Kramer post hoc test to compare the effect of the increasing CD concentrations on the cell surface LAMP1. Asterix represents significant changes (* *p* < 0.05) compared to the 100% LAMP1 expression level. (**B**) Proportion of LAMP1 protein on the cell membrane of 3T3-MDR1 cells compared to the proportion of parental NIH-3T3 cells for 20 min 5 mM CD treatment (low). Bars represent the mean ± SEM values of LAMP1 protein on the cell surface from at least three experiments displayed as a percentage of the total LAMP1 protein level. A one-way ANOVA was performed with Tukey’s HSD Test for multiple comparisons to compare the effect of the cholesterol depletion on the cell surface fraction of the cellular LAMP1. Asterix represents significant changes at *p* = 0.05 compared to the normal LAMP1 expression levels in the absence and the presence of calcium, respectively. (**C**) Photomicrographs of NIH-3T3 and 3T3-MDR1 cells following cholesterol depletion. Fluorescence intensities were only slightly higher than background signals in NIH-3T3 cells. The upper panels are fluorescence images of the 5 mM CD-treated samples of NIH-3T3 and 3T3-MDR1 cells in the presence of EC Ca^2+^. The lower panels are bright-field images of the same cells. The scale bar represents 50 μm.

Cholesterol-reducing CD treatments stimulated membrane repair [66,81,82]. In membrane repair, damaged PM parts are removed and replenished by typical trajectories of exocytosis [68,83]. The role of lysosomal exocytosis in a calcium-dependent manner is a well-appreciated phenomenon in membrane repair [68]. It was suspected that cholesterol extraction permeabilizes the PM to small ions in the extracellular milieu, such as Ca^2+^, and triggers lysosomal exocytosis [66]. In addition, the P-gp-expressing MDR cells are often associated with increased volume and the number of endosomes, probably including lysosomes [69,84], and have enhanced membrane turnover [16,85,86]. These facts led us to hypothesize that the enhanced exocytosis of P-gp was likely to indicate such mechanisms. In CD treatment, homeostatic compensatory responses via exocytosis could originate from vesicles of high cholesterol content, like pools of recycling PM, Golgi, late endosomes, and lysosomes. To prove our idea, the lysosome-associated membrane protein 1 (LAMP1) cell surface level was measured, a marker for lysosomal and late endosomal exocytosis. LAMP1 increased proportionally with decreasing cholesterol by applying increasing CD concentrations in the presence of extracellular Ca^2+^ in P-gp-overexpressing 3T3-MDR1 cells (Figure 4A). The effect was significant in the case of the MDR1 cells without calcium as well, but it was more pronounced in the presence of extracellular Ca^2+^. Another report showing that many MDR cell lines secrete increased lysosomal enzymes [70] prompted us to determine if this relates to our observed effects.

The 3T3-MDR1 cell line stably expressing Ppg was compared to its parental non-transfected NIH-3T3 for LAMP1 expression. Both cell lines expressed LAMP1 (Figure S2). An increased proportion of LAMP1 was present on the surface of 3T3-MDR1 cells (7.8%) as compared to non-transfected cells (2.8%) in the absence of added Ca^2+^ and the presence of Ca^2+^ (9.4% compared to 4.7%; Figure 4B) added extracellularly. Cholesterol depletion significantly enhanced cell surface levels from 7.8 to 14.3% in the absence of added Ca^2+^ and from 9.4 to 18.4% when Ca^2+^ was present in the buffer. In parental NIH-3T3 cells, a minor enhancement was observed, with cell surface LAMP1 increasing from 2.8 to 4.3% in the absence of Ca^2+^, and there was a significant change from 4.7 to 5.6% in the presence of Ca^2+^. Another lysosomal marker, LAMP2, was also measured. Figure 4C shows representative images of the increased cell surface appearance of the LAMP2 after 5 mM CD treatment in NIH-3T3 and 3T3-MDR1 cells in the presence of Ca^2+^. These data suggest increased recycling between lysosomes and the PM in P-gp-overexpressing MDR cells.

Moreover, lysosomal exocytosis, which takes part in membrane repair, was further enhanced in P-gp overexpressing cells after cholesterol depletion of the PM. Our data indicate that extracellular calcium, as it was known earlier, and PM cholesterol, as a new observation, have a role in membrane repair. Additionally, it suggests that cells that have a larger pool of lysosomes have enhanced membrane repair and might have less sensitivity to membrane damage. Even P-gp might have a role in these processes.

### 2.6. Enhanced Number and Increased Stability of Lysosomes in 3T3-MDR1 Cells

**Figure 5:**
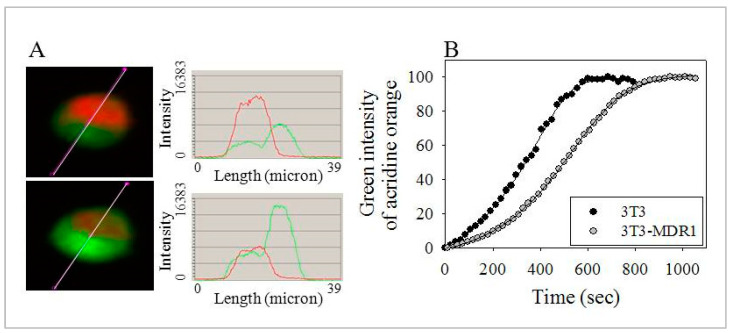
3T3-MDR1 cells have more stable lysosomes than parental NIH-3T3 cells. The blue-light-induced photo-destruction of acridine orange (AO) was investigated using laser-scanning cytometry. (**A**) AO accumulating in the lysosomes shows red fluorescence as crowded molecules are stacked at high concentrations. AO is green at lower concentrations, where molecules are monomeric (upper (**A**): before illumination; lower (**A**): after illumination). On the left side of (**A**), white lines across the cells indicate the location of the red–green intensity profile plotted on the right side. During the illumination, AO damages lysosomal membranes, and the dye molecules are released from the acidic lysosomal milieu, decreasing red and increasing green fluorescence. (**B**) Green intensity changes from a representative experiment. The time required to reach half-maximum fluorescence of the green fluorescence intensity in 3T3-MDR1 cells (410 ± 180 s) was significantly longer than in NIH-3T3 cells (300 ± 140 s) calculated from five separate experiments (*p* < 0.05 by unpaired, one-tail *t*-test).

To further investigate the role of lysosomes in this membrane repair process that depends on the fusion of lysosomes with the PM, the number and the stability of lysosomes in the cells were tested. First, we compared the number of acidic vesicles in the P-gp transfected and non-transfected cell lines by measuring the accumulation of a lysosomotropic weakly basic chromatic dye, neutral red (NR). By light microscopy, 3T3-MDR1 cells showed more intense NR staining (Figure S3A,B). Next, NR intensity was measured by quantitative imaging cytometry, and 3T3-MDR1 cells accumulated 1.5 times more NR than NIH-3T3 cells (Figure S3C). Flow cytometry also determined these cells’ light side scatter (SSC) intensities. This measurement also indicated that 3T3-MDR1 cells have 1.5 times more granularity, proportional to the number of vesicles in single cells (Figure S3D). These data signify more numerous and functional endo-lysosomes in 3T3-MDR1 cells than in their parent NIH-3T3 cell line [87]. Following this, the stability of the lysosomes was examined by measuring the photo-oxidation of another weakly basic lysosomotropic dye, acridine orange (AO) [88], using laser-scanning cytometry. AO is excited by blue light, acts as a metachromatic dye, and emits red fluorescence when highly concentrated inside endo-lysosomes. AO fluoresces in green when it dilutes into the cytosol because of the rupturing of lysosomal membranes induced by the blue light-activated AO causes photo-oxidative damage to the endo-lysosomal membranes (Figure 5A) [89]. The initial difference in dye accumulation in lysosomes, determined by red fluorescence, agreed with the previously measured neutral red accumulations for the two cell lines. The loss of lysosomal integrity was monitored by measuring the time required to reach half the maximum of the green fluorescence intensity from the beginning of the blue 488 nm laser illumination. It was found that the 3T3-MDR1 cells exhibited longer half maximum time (409 ± 184 s) compared to the NIH-3T3 cells (296 ± 135 s) (Figure 5B). These laser-scanning cytometric measurements indicated more stable lysosomes in the MDR cells. Our data provided evidence for the increased number and stability of endo-lysosomes in 3T3-MDR1 cells, and these data further suggest that 3T3-MDR1 cells might have enhanced membrane repair and consequently less sensitivity to membrane damage than NIH-3T3 cells.

### 2.7. Lysosomes of 3T3-MDR1 Cells Were More Resistant to Photolysis Than Lysosomes of Parental NIH-3T3 Cells, even after Cholesterol Depletion

**Figure 6:**
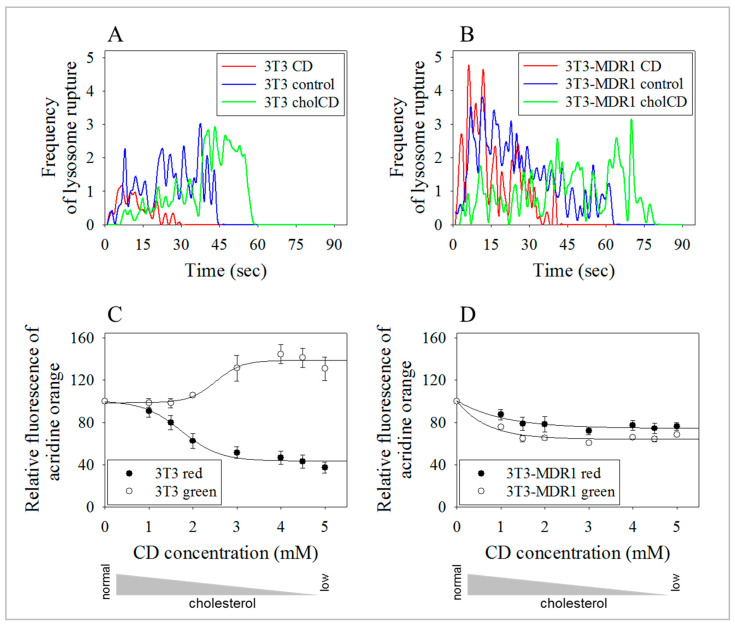
Effect of cholesterol modulation on the stability of lysosomes. The stability of lysosomal membranes was determined by acridine orange (AO) photo-destruction in NIH-3T3 and 3T3-MDR1 cells. (**A**,**B**) indicate the frequency of lysosome ruptures per cell with time in NIH-3T3 ((**A**) CD 29 s; Control 46 s; chol-CD 60 s) and 3T3-MDR1 cells ((**B**) CD-40 s; Control-65 s; chol-CD-79 s). Representative curves for 3 mM cyclodextrin-treated cells are shown. A higher dose of 5 mM CD treatment destroyed lysosomes before irradiation in NIH-3T3 cells, probably by the ambient light (Supplementary Videos S1 and S2). (**C**,**D**) Dual wavelength monitoring of the rupture of lysosomes in NIH-3T3 (**C**) and 3T3-MDR1 (**D**) cells with increasing concentrations of CD. Red and green steady-state AO fluorescence was determined using a plate reader, avoiding intense blue light exposure. Values represent mean ± SEM values from four experiments.

Cholesterol likely plays an essential role in maintaining lysosomal membrane integrity [90,91,92]. It was confirmed that cholesterol depletion with CD decreased lysosomal membrane stability, although the experimental setup included a 24 h chase step after CD treatment [93]. The lysosomal membrane stability of our two cell lines was examined at the earliest following cholesterol modulation using live-cell video microscopy. Lysosome stability was affected by cholesterol modulation in both cell lines, with cholesterol depletion reducing the exposure times needed for photolysis and added cholesterol increasing it (Figure 6A,B). Representative curves of measurements for the frequency of the rupture of photo-damaged lysosomes in cells treated with 3 mM cyclodextrin are shown in Figure 6. The higher dose of 5 mM CD resulted in the rapid elimination of lysosomes from the NIH-3T3 cells that were already at ambient light conditions (Supplementary Videos S1 and S2). To determine the concentration dependence of cholesterol depletion more precisely, we examined lysosomal photolysis using a spectrofluorometric plate reader. CD treatment sensitized more NIH-3T3 cells to AO, as they exhibited a steeper decline in red fluorescence and enhanced green fluorescence with increasing levels of cholesterol depletion (Figure 6C). This corresponds to the disappearance of red acidic granules and conversion of the dye into the monomeric green form in cytoplasmic and nuclear compartments. These findings agreed with our live video microscopy results. A different pattern was observed with 3T3-MDR1 cells with a slight decrease in both red and green fluorescence, a sign of a reduction in the number of acidic organelles (Figure 6D).

While the changes in fluorescence indicated the intracellular rupturing of lysosomes in the NIH-3T3 cells, the alterations in the 3T3-MDR1 cells indicated a different mechanism for the loss of lysosomes. Based on our LAMP1 and LAMP2 observations, the latter was likely the result of lysosomal exocytosis. These data reverberate the notion that 3T3-MDR1 cells have more efficient membrane repair than NIH-3T3 cells.

### 2.8. The More Effective Repair Mechanisms of 3T3-MDR1 Cells Result in Higher Viability after Cholesterol Modulation

**Figure 7:**
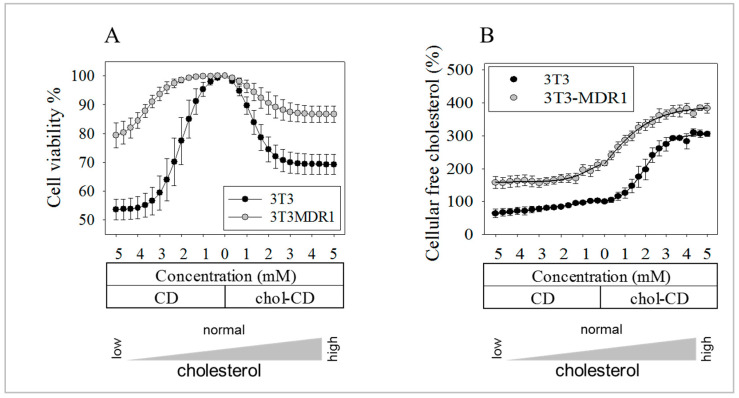
Cell viability and cholesterol content of NIH-3T3 and 3T3-MDR1 cells. Cell viability (**A**) and free cholesterol concentrations (**B**) in NIH-3T3 and 3T3-MDR1 cell populations with various concentrations of CD and chol-CD. (**A**) Cholesterol perturbation of the PM decreased the viability of parental NIH-3T3 cells more than that of MDR1 cells, comparing the 5 mM CD and 5 mM chol-CD samples (*p* = 0.0086 and *p* = 0.0020 by unpaired, one-tail *t*-test). Values represent the mean of three independent experiments with 95% confidence intervals. (Curve fitting of individual raw data is exemplified in Figure S4). Statistical analysis was performed to compare primary data of 5 mM CD, 5 mM chol-CD, and control samples (0 mM CD) in both cell lines. Results for pairwise comparisons are the following: 3T3 CD versus Control: *p* = 0.0007; 3T3 Control versus chol-CD *p* = 0.0020; 3T3-MDR1 CD versus Control *p* = 0.0260; 3T3-MDR1 Control versus chol-CD *p* = 0.0104 by paired, one-tail *t*-test. (**B**) MDR1 cells contain significantly more cholesterol than parental NIH-3T3 cells *p* < 0.0001 by unpaired, one-tail *t*-test. Free cholesterol was measured using filipin in flow cytometry. Values represent mean ± SEM of three independent experiments. Statistical analysis was performed between the 5 mM CD and 5 mM chol-CD samples and control samples (0 mM) in both cell lines. 3T3 CD—Control *p* = 0.0132; 3T3 Control—chol-CD *p* < 0.0001; 3T3-MDR1 CD—Control *p* = 0.0033; 3T3-MDR1 Control—chol-CD *p* < 0.0001 by unpaired, one-tail *t*-test.

Better membrane repair should lead to better cell survival after cholesterol modulation. Therefore, our P-gp-expressing cell line was compared with its parental line focusing on sensitivity to cholesterol perturbations. Cholesterol depletion reduced the cell viability of the NIH-3T3 control cells by 46 ± 4% following treatment with 5 mM CD (Figure 7A). This is compared to (less than half of) the 21 ± 4% decrease in the P-gp expressing line. Interestingly, adding cholesterol to the PM resulted in similar decreases in cell viability. The cholesterol content of these cells was measured by filipin staining and flow cytometry (Figure 7B). The P-gp overexpressing line had higher levels of cellular cholesterol (220.4 ± 13%) compared to its counterpart (100 ± 6%).

To see if P-gp transfection would be responsible for cell viability increasing in another cell line, measurements were repeated in MDCK cells (Figure S5). MDCK-MDR1 cells had significantly higher viability in the range of cholesterol disturbance between 0–2 mM CD cholesterol extraction and 0–2 mM chol-CD supplementation ranges. However, differences in canine cells were minor (approximately 3%) and showed different patterns compared to NIH mouse cells. In MDCK cells with increasing cholesterol levels, differences decreased.

These experiments proved that a single gene, human P-gp transfection is responsible for the resistance against cholesterol perturbations in these immortal cells.

## 3. Discussion

The best-understood mechanism for maintaining steady-state cholesterol levels is cholesterol synthesis [78]. Excess cholesterol in the PM blocks synthesis because of an increased redistribution of cholesterol into intracellular (IC) pools, whereas depletion of PM cholesterol results in the activation of genes that facilitate production. Newly synthesized cholesterol either bypasses the Golgi complex [94,95] or follows the secretory pathway and is incorporated into the raft structures [96,97]. Through the generation of these microdomains, cholesterol exhibits a vital role in sorting and distributing proteins [98], including P-glycoprotein [99], to the cell surface [74]. Depletion of PM cholesterol by cyclodextrins leads to the activation of a sterol sensor and transporter (OSBP) in addition to a ceramide transporter (CERT) between the ER and the trans-Golgi [77]. Activation of these two transporters intensifies the nucleation of the raft domains in the Golgi, and the appearance of newly synthesized cholesterol in the PM begins to occur after 10 to 30 min [96]. The increase in raft-associated UIC2 reactive P-gp levels that we have observed were compatible with the timing of these compensatory mechanisms after cholesterol depletion. Cholesterol redistribution into IC pools correlates with the delivery of UIC2 reactive P-gp, like the increased endocytosis of caveolae after cholesterol enrichment already described [100,101]. Increasing cholesterol content downregulated the exocytosis of this conformer, similar to the decrease in cholesterol export through classical secretory pathways. Analogous secretory changes may explain the cholesterol loading-induced block in the exit of the VSVG protein from the trans-Golgi network (TGN) [102]. On the other hand, cholesterol depletion significantly intensified the exocytosis of the UIC2 binding P-gp to the PM, like compensatory cholesterol delivery.

The role of exocytosis in the cell surface increase in this conformer after CD treatment has been confirmed using chemical inhibitors. Exo1 decreased the cell surface level of UIC2 binding P-gp under all conditions. The extent of inhibition was cholesterol-dependent and proportional to exocytosis induced by cholesterol modulations. The disturbance of both trafficking systems in the presence of BFA only slightly decreased the cell surface level of the UIC2 binding conformers in the CD-treated samples. From these experiments, it looks likely that the exocytosis of raft-associated UIC2-binding P-gp [15,50,62,63] is cholesterol-dependent and closely linked to the compensatory cholesterol and sphingolipid export via the secretory pathway. Lipid environment and cholesterol have a direct effect on P-gp’s conformations [64] and ATP hydrolysis [103,104]; also, it is involved in its exporter function [45,58,105], and several human studies indicating a link between body cholesterol levels and mdr1 gene polymorphism underline the importance of our findings [106]. 

Treatment with CD stimulates other exocytotic events that are important in membrane repair, for example, exocytosis of lysosomes in a Ca^2+^-dependent manner. Lysosomal exocytosis, measured by the appearance of LAMP1 and LAMP2, occurred in our P-gp-overexpressing cell line after CD treatment. Similar changes were observed when measuring the β-hexosaminidase activity in the media [66]. The level of LAMP1 increased gradually with the extent of cholesterol depletion. Extracellular Ca^2+^ enhanced the process; however, it also occurred when the extracellular buffer did not contain Ca^2+^, underlying the cholesterol dependence of the phenomenon. Fusion events require Ca^2+^, which is released from the lumen of the fusing organelles [107]. In the case of lysosome exocytosis, the two involved “organelles” are the PM and the lysosomes. This means that the increase in intracellular Ca^2+^, which is required for the lysosome and PM fusion, may originate from the EC milieu or the lysosome itself. Recently, an inwardly rectifying cation channel, the mucolipin transient receptor potential (TRP) channel 1 (TRPML1), has been confirmed to be responsible for intra-lysosomal-Ca^2+^ release and the regulation of lysosomal exocytosis [108,109,110]. Interestingly, the channel was demonstrated to be under significant lipid control [111].

The increased volume of the endo-lysosomal compartment upon the development of MDR caught our attention because of its great capacity for sequestering significant amounts of weak base chemotherapeutic drugs. Such enlargement can also be observed in our 3T3-MDR1 cell line (Figure S3A–C). In agreement with these observations, our observation of the increased flow cytometric side scatter (SSC), which reflects the granularity or internal complexity of cells, also indicates the expansion of the vesicular system in 3T3-MDR1 cells (Figure S2D). An increased rate of lysosomal exocytosis and enhanced membrane turnover is also a unique feature of MDR1-expressing cells. Thus, the fusion of lysosomes with the PM during cell injury might provide additional benefits to these cells in membrane repair. An almost two-fold increase was found in the cell surface appearance of the lysosomal marker in the 3T3-MDR1 cells in contrast to a minor increase in the NIH-3T3 cells (Figure 4B). This suggests that the rate of lysosomal exocytosis is already increased for 3T3-MDR1 cells and can further be enhanced after cholesterol depletion.

In addition to substantial lysosomal proteins like LAMP or Hsp70 [112,113], lysosomal cholesterol has been reported to directly control the permeabilization of lysosomal membranes [114,115,116,117]. PM cholesterol, often increased during MDR, is likely to influence lysosomal cholesterol content [93] and, consequently, the membrane stability of, e.g., the 3T3-MDR1 cells. This cell line is usually cultured under conditions to maintain drug selection, as most other cell lines overexpress drug-pumping ABC transporters. 3T3-MDR1 cells indeed exhibit twice as much cellular cholesterol as NIH-3T3 cells. Cholesterol perturbations induced similar changes in the cholesterol content of both cell lines; however, they revealed remarkable differences in viabilities (Figure 7A,B). We assumed that the membrane repair of 3T3-MDR1 cells after cholesterol depletion might carry more rafts and cholesterol to the cell surface, in accordance with the increased exocytosis of the raft conformer P-gp. In case of cholesterol overload, endocytosis of the cell surface rafts to intracellular cholesterol reservoirs manages absorption of cholesterol in excess. These data suggest an enhanced cholesterol compensatory and repair mechanism in 3T3-MDR1 cells. A two-fold more significant increase in cell surface LAMP proteins reflects robust lysosomal exocytosis after CD treatment. The lysosomal membrane photo-destruction experiments indicated that cholesterol depletion ruptures fewer lysosomes in 3T3-MDR1 cells.

Furthermore, it appeared to trigger the fusion of lysosomes with the PM in these cells, contrary to the NIH-3T3 cells. We recognized that the stability of lysosomes is influenced by acute modulations of PM cholesterol in minutes (Figure 6) and not only on the longer time scale of hours or days, as described in [93]. These endo-lysosomal and repair differences were manifested in cell viability differences, measured by the propidium iodide permeability of the PM, which is the primary target of cholesterol extraction.

Lysosomes are dynamic organelles that fuse with late endosomes, the PM, phagosomes, and autophagosomes [83]. Any interference with the trafficking of lysosomes largely influences their stability [118,119,120,121]. One possible explanation for this observation is that the stability of lysosomes requires continuous fusions with other organelles and PM [122]. The cholesterol content of lysosomes is approximately 6% of the cellular content due to the continuous circulation of cholesterol between lysosomes and the PM [123]. Our results also demonstrated that lysosome stability quickly responds to PM cholesterol levels. Therefore, it can be assumed that if a cell effectively controls PM cholesterol, it might also influence lysosome cholesterol levels. Our results show that the extent of cholesterol loss from lysosomes might dictate life and death, whether it acts as initial feedback for membrane repair or enhances the suicidal membrane permeability of lysosomes.

MDR cells have more effective cholesterol compensatory mechanisms, including increased uptake and esterification of excess cholesterol from PM [124,125] and enhanced cholesterol synthesis [126]. The enhanced internalization of raft domains constitutes another cholesterol pool. Moreover, the higher number of raft domains that contain most cholesterol with low “escape tendency” in the PM [127] also gives MDR cells the ability to react with relatively small changes in cholesterol level. Our cell viability measurements following cholesterol addition also show that MDR cells are more proficient in managing increased PM cholesterol levels. The more effective membrane cholesterol homeostatic and membrane repair process observed in P-gp-overexpressing MDR cells includes the re-formation of raft domains to the PM indicated by the rise in the raft-associated conformer of P-gp. Even though P-gp overexpression appears to be an essential difference between 3T3-MDR1 and NIH-3T3 cells and MDCK-MDR1 and MDCK cells, the direct contribution of P-gp to cholesterol homeostasis is still as questionable as its involvement in stabilizing membrane microdomains. Alternative MDR mechanisms and P-gp expression, however, are likely interdependent. Cells that display effective membrane turnover likely exhibit efficient protein turnover as well.

## 4. Materials and Methods

### 4.1. Cell Culture

Most experiments used the human mdr1 gene-transfected NIH-3T3 mouse-origin cell line (NIH-3T3 MDR1 G185 cells, gift from M. Gottesman). This spontaneously immortalized mouse fibroblast embryonic cell line (CRL-1658 at ATCC, Gaithersburg, MD, USA) is stably transfected by a retroviral vector carrying the mdr1 gene [128,129]. Cells were cultured at 37 °C in a humidified gas incubator containing 5% CO_2_ and subdivided every third day in Dulbecco’s modified Eagle’s medium (DMEM) supplemented with 10% heat-inactivated fetal calf serum, 2 mM L-glutamine, 25 µg/mL gentamycin, 670 nM doxorubicin. Two–three days before the experiments, the doxorubicin-free medium replaced the medium. NIH-3T3 mouse fibroblast cells, as the non-transfected parental counterpart of P-gp-expressing cells, were also used in some of the experiments. Cells were tested for mycoplasma regularly. The canine-origin spontaneously immortalized adult-kidney MDCK epithelial cell line (CCL-34 at ATCC, Gaithersburg, MD, USA), and its human Pgp-transfected pair, the MDCK-MDR1 cell line, was a gift from Balázs Sarkadi (member of the Hungarian Academy of Sciences). The canine-origin cells were maintained in similar conditions to the mouse cells.

### 4.2. Chemicals, Antibodies

Cell culture media, supplements, Exo 1 (2 (4 Fluorobenzoylamino)-benzoic acid methyl ester), brefeldin-A, and other chemicals, unless specified, were purchased from Sigma (Budapest, Hungary). CD (random methyl β-cyclodextrin, >95% purity) and chol-CD (cholesterol inclusion complex of CD, cholesterol content 3.0–5.1%, determined by HPLC) were a kind gift from Cyclolab Ltd. (Budapest, Hungary). UIC2 (IgG2a, HB-11027, [130]) and 15D3 (IgG1, HB-11342, [131]) mouse monoclonal antibodies (mAb) produced against human P-glycoprotein were isolated from the supernatant of their hybridomas (from ATCC, Gaithersburg, MD, USA) and purified using protein A affinity chromatography. The mAb preparations were >97% pure, checked by SDS-polyacrylamide gel electrophoresis.

### 4.3. K_d_ and B_max_ Values of Antibodies, Cell Membrane Cholesterol Modulations

Cell monolayers at near confluence were harvested by trypsin treatment for 3 min (0.05% trypsin and 0.02% EDTA in phosphate-buffered saline (PBS), pH 7.4) and washed twice with PBS supplemented with 8 mM glucose (glucPBS). The pellets were suspended at a concentration of 1 × 10^6^ cells/mL and incubated with 5 mM CD, or 5 mM chol-CD, or as a control sample without it for 20 min at 37 °C and washed twice again with glucPBS at room temperature (modulation of cell membrane cholesterol). Then cells were labeled with various concentrations of 15D3 mAb tagged with A647 (15D3-A647) or UIC2 mAb labeled with A647 (UIC2-A647) for 30 min at 37 °C, followed by two final washes of ice-cold PBS. Samples were measured by flow cytometry (BD FACSArray, Budapest Flow Soft Kft.) in the presence of propidium iodide (PI) to exclude dead cells. Experiments were repeated at least 3 times. Curve fitting was conducted by Sigma Plot version 10.0 software (Systat Software, Inc. San Jose, CA, USA). Dissociation constants (K_d_), and the fluorescence of total antigen binding capacities of antibodies (B_max_) were calculated according to the saturation model of ligand binding to one site corrected for non-specific binding using the f = B_max_ × abs(x)/(K_d_ + abs(x)) + Ns × x) equation. Values are given as mean ± Standard Deviation (SD).

### 4.4. Endocytosis Measurements

Cells were labeled in glucPBS (0.25 million cells/0.1 mL) with UIC2-A647 or 15D3-A647 (100 µg/mL, 6.67 × 10^−7^ M) at 37 °C for 30 min. Next, modulation of cell membrane cholesterol was applied, and cells were incubated in DMEM supplemented with 1% heat-inactivated fetal calf serum, 2 mM L-glutamine, and 25 µg/mL gentamycin, and 10 mM Hepes, pH 7.4 for different time periods (0, 15, 30, 45, and 60 min). The medium was replaced for glucPBS, and cells were divided into two aliquots. In one, the surface-bound antibody was removed by acidic treatment (0.1 M glycine, 0.5 M NaCl, pH 2.5) for 3 min at room temperature (RT), while the other aliquot was kept in PBS. Finally, cells were washed twice, and 5 µg/mL PI was added and assayed using a flow cytometer (BD FACSArray from Soft Flow Kft, Budapest, Hungary). PI was excited at 542 nm and detected in the Yellow channel (585/42 nm), while A647 was excited at 635 nm and measured in the Red channel (661/16 nm).

Data analyses: First, the PI-positive fraction was excluded, and values were corrected for non-specific background. Acid-wash-treated cells estimated the amount of internalized P-gp, and PBS-treated cells provided estimates for total surface-bound and internalized transporters. The degree of internalization was calculated as the ratio of the antibody labeling of the acid-treated and untreated cells as a percentage. Kinetic curves for endocytosis were generated. The effect of cholesterol depletion and enrichment was determined at the 15 min time point compared to the control sample, where cholesterol was unaltered (mean ± SD).

### 4.5. Exocytosis of the UIC2 Reactive Fraction

Cells were incubated in gluPBS with unlabeled UIC2 mAb (100 µg/mL) for 30 min at 37 °C and washed twice with ice-cold PBS to pre-cover cell surface P-gp with unlabeled mAb. Then, cholesterol modulations were applied, and subsequently, cells were incubated in DMEM (supplemented as described above) for different durations (30, 25, 20, 15, 10, 5, and 2 min) in the presence of A647 conjugated UIC2 mAb (100 µg/mL) to monitor the newly upcoming P-gp. Cells were washed twice with ice-cold PBS and measured by flow cytometry.

Data analyses: PI-positive population was excluded. To calculate the kinetics of exocytosis, the half-labeling time of the UIC2-A647 mAb was considered regarding short labeling times in some samples. The effect of cholesterol depletion and enrichment for the exocytosis in percentage was determined at the 15 min time point compared to the control sample, where cholesterol was unaltered (mean ± SD).

### 4.6. Inhibition of Exocytosis

Cells were pre-incubated with Exo 1 (2-(4-Fluorobenzoylamino)-benzoic acid methyl ester, 400 µM) or brefeldin-A (360 µM) for 2 h in glucPBS at 37 °C. Then, cells were treated for cholesterol modulations, and the UIC2 reactive cell surface P-gp were labeled with 100 µg/mL UIC2-A647 for 5 min, washed once with ice-cold glucPBS, and measured in the presence of PI. To use a shorter labeling time, a 5 min pulse label was applied, which labels 91.7 ± 3.7% (mean ± SD) of P-gp compared to 30 min of labeling in untreated samples.

### 4.7. Cell Surface Appearance of LAMP1 and LAMP2 after Cholesterol Extraction

Cell monolayers of the NIH-3T3 mouse fibroblast cell line and its human mdr1-transfected counterpart were harvested as described previously and washed either with phosphate-buffered saline (PBS) or with Hank’s Balanced Salt Solution (HBSS containing 1 mM CaCl_2_) supplemented with 8 mM glucose. In the case of the LAMP2 samples, experiments were carried out with PBS supplemented with 1 mM EDTA. The cell pellets were suspended and incubated with or without CD. Next, cells were labeled with 2.5 µg/mL anti-mouse LAMP1-PE (CD107a-PE) or 10 ug/mL LAMP2-Alexa488 (CD107b) for 30 min at 37 °C, washed, and immediately fixed with freshly prepared 2% paraformaldehyde (PFA) for 1h at RT. To determine the total LAMP levels, cells were fixed first with 2% PFA for 20 min at RT, washed, and quenched with 1.5 mg glycine/mL solution for 10 min. Anti-mouse LAMP1-PE antibody or LAMP2-Alexa488 was added for 1 h at RT in 0.5% saponin/ 1% BSA buffer. Cells were washed extensively with 0.5% saponin/ 1% BSA buffer, resuspended in PBS 1% BSA, and measured immediately by flow cytometer (BD FACSArria III). Cell surface anti-mouse LAMP fluorescence intensities are expressed as a percentage of a cell’s total LAMP fluorescence.

### 4.8. Measurement of Cellular Cholesterol Levels and Cell Viability of MDR1 Transfected and Non-Transfected Cell Lines

The NIH-3T3 mouse fibroblast cell line and its human mdr1-transfected counterpart were used in these experiments. Cells were treated with different concentrations of CD or chol CD, as is common practice, and washed twice with glucPBS. Cells were either labeled with PI (5 µg/mL) and measured immediately by flow cytometer or fixed with freshly prepared 3% PFA for 1h at RT for further procedures. Fixed cells were washed twice with PBS and incubated with 1.5 mg glycine/mL PBS for 10 min at RT. Cells were finally stained with 125 µg/mL filipin in PBS / 10% FBS for 1 h at RT in the dark and measured by flow cytometer (BD FACSArria III). PI fluorescence signals were taken at 561 nm excitation with a 561LP filter, while a 450/40BP filter was used with 375 nm excitation to detect filipin signals. Measured cholesterol contents were corrected for cell volume. Cell viability curves were calculated according to sigmoidal dose–response ligand binding using the

f = min + (max − min)/(1 + 10^(logEC50-X)^)

equation with Sigma Plot version 10.0 software.

### 4.9. Determination of Lysosome Content of Cells

Lysosomotropic chromatic dye neutral red (NR) staining was used for quantitative imaging cytometry. For single-cell analyses, cell nuclei were stained by Hoechst 33342, and lysosomes were loaded with NR. Cells were recognized and contoured by a total cellular signal, adding together the Hoechst 33342 (excited with 405 nm violet laser and detected via 430–470 nm bandpass filter) and NR fluorescence (excited with 488 nm blue laser and detected via a 650 nm long-pass filter) and the light absorbance of NR (illuminated at 488 nm). Single cells were defined by their area and DNA content using the Hoechst 33342 signal. Finally, the lysosomal content of single cells was determined by the NR chromatic intensity of identified cells. Measurements and analyses were made by applying the fluoro-chromatic option of our iCys laser-scanning imaging cytometer and its iNovator Applications Development Toolkit 7.0 software (Thorlabs Imaging Systems, Sterling, VA, USA, formerly known as CompuCyte).

### 4.10. Measurements of Photo-Destruction of Lysosomes

Acridine orange (AO) sensitized light-induced photo-destruction of lysosomes was investigated by laser-scanning cytometry (iCys). Cells were cultured in 8-well tissue culture plates in DMEM and incubated until they reached confluency. Cells were stained with 1 µg/mL AO in PBS for 30 min at 37 °C and washed twice with PBS. AO-loaded cells were then illuminated by a solid-state 488-nm blue laser in an area repeatedly in 50 cycles by 40× objective (Olympus, UIS2 40× plan fluorite, NA 0.75). The area was set to be covered by 2 × 2 scanning field windows using 0.25-μm step size (one scanning field window is 1024 × 768 steps). Fluorescence intensity of red (565–595, 580/30 nm) and green (515-545, 530/30 nm) ranges of light emission was measured at each time point. Data were exported from the iCys 7.0 software to the Microsoft Excel spreadsheet program, and calculations were carried out. The high concentration of stacked AO dye molecules accumulating in lysosomes fluoresces red. During blue light illumination, the AO-sensitized lysosomal membrane ruptures, dye molecules are released from the acidic milieu, and the red fluorescence decreases. Continuous release of AO dye to the cytosol and its binding to DNA in the nucleus increases green fluorescence. The time constant at which the green fluorescence is half of the total green fluorescence intensity of cells was determined by fitting a sigmoid curve to data by applying an online curve-fitting tool (http://zunzun.com/ (accessed on 1 March 2014), recently http://www.findcurves.com/). The mean and SD of the time constant in seconds of the half-maximum-fluorescence were calculated from 15 and 10 cells. Cholesterol dependence of photo-destruction of lysosomes was investigated as described henceforth. The 20 min acute cyclodextrin (CD; chol-CD) treatment of adherent cells was followed by the 30 min acridine orange (AO) incubation, and then a field of view of cells was exposed to a continuous blue light irradiation (FITC filter set) using the epifluorescence Hg-lamp illumination of the iCys system (an Olympus IX-71 fluorescent microscope). Microscopic videos were recorded by an Olympus DP71 (Olympus, Budapest, Hungary) high-resolution color digital camera from the beginning of irradiation. Events of lysosomal rupture corresponding to fluorescence flashes in the green channel were recorded over time.

### 4.11. Analysis of Image Series

The detection of exocytotic events was performed using an automated program based on what had been used for the analysis of calcium sparks on time series confocal images [132]. In the first step, inter-mean thresholding with a correction factor of 1.65 was performed on the first frame in each series to create a mask of pixels belonging to cells. Morphological closing was applied on this mask to eliminate features more minor than 5 pixels in diameter. A background area utterly free of cells was manually selected, and the average intensity of this area was subtracted from all pixel intensity values. F/F0 series were calculated for each pixel, where intensities below mean + 1.8 SD of the time series data for the given pixel were considered baseline (F0). Events were identified on each frame as contagious groups of pixels with intensity values above mean + 1.12 SD containing at least one pixel above mean + 1.18 SD within the cell area mask. Events containing less than 45 pixels were dropped. The diameter of this group of pixels was measured in the X and Y direction, and the arithmetic mean of these two numbers was used to describe the size of each exocytotic event. The number of events on all images was counted to determine the event frequency for a given condition. Events on consecutive images at the exact location were counted as a single event. Numbers were normalized to the cell number. Irradiation times required to destroy every lysosome were also presented for both cell lines.

### 4.12. Cholesterol Extraction Effect on Lysosomotropic Dye Accumulation

For dye accumulation studies, cells were cultured in 96-well tissue culture plates at a density of 1 × 10^5^ cells/well in DMEM. Cells were incubated at previously described adequate conditions for 24 h. According to previous observations, this incubation period allows cell recovery, adherence, and exponential growth [87]. After gently replacing the medium, cells were incubated with different concentrations of CD solution and 1 µg/mL acridine orange solution, and fluorescence intensity measurements were taken with a Synergy HT reader (Bio-Tek Instruments, Winooski, VT, USA) at 460 nm excitation and 645 nm emission for red fluorescence intensities, and at 485 nm excitation and 528 nm emission for green ones.

## 5. Conclusions

The cholesterol depletion-induced injury of the PM provoked exocytotic events. These include lysosomal exocytosis and the delivery of raft domains to the cell surface, indicated by increased exocytosis of the raft conformer P-glycoprotein. Cyclodextrin-elicited membrane repair was more effective in P-gp expressing 3T3-MDR1 cells than in its parental NIH-3T3 mouse-origin fibroblasts cells. This phenomenon brings about significantly better cell viability in MDR1 cells after cholesterol modulations of the plasma membrane. The increased cell viability for cholesterol modulations was reproduced in human P-gp gene-transfected canine-origin epithelial cells. The more stable, expanded endo-lysosomal system and the higher cholesterol level provide durable membrane cholesterol homeostasis of the multidrug-resistant P-gp overexpressing cells, which play a crucial role in this process. There is a definite link between multidrug resistance and P-gp expression-related cellular cholesterol increase and the durability of cell membranes in these spontaneously immortalized cells. However, to what extent we may attribute the P-gp expression or other factors to induce modified lipid metabolism in human cancer cells is still a question. The notion that P-gp might have a specific role in the membrane stabilization of lysosomes requires further experimentation.

## Data Availability

The data presented in this study are available in this article and its supplementary material.

## Supplementary Materials

The following supporting information can be downloaded at: https://www.mdpi.com/article/10.3390/ijms241512335/s1.
