# Supplementary material for: Cholesterol-Depletion-Induced Membrane Repair Carries a Raft Conformer of P-Glycoprotein to the Cell Surface, Indicating Enhanced Cholesterol Trafficking in MDR Cells, Which Makes Them Resistant to Cholesterol Modifications
Source: Int J Mol Sci. 2023 Aug 2;24(15):12335. doi: 10.3390/ijms241512335 (PMC10419235; doi:10.3390/ijms241512335)
Supplement: Supplementary file 1 [file ijms-24-12335-s001.zip › ijms-2510443-supplementary.pdf]

SUPPLEMENTARY FIGURES AND LEGENDS

Cholesterol-Depletion-Induced Membrane Repair Carries a Raft Conformer of P-Glycoprotein to the Cell Surface, Indicating Enhanced Cholesterol Trafficking in MDR Cells, Which Makes Them Resistant to Cholesterol Modifications

Zsuzsanna Gutay-Tóth [1,2], Gabriella Gellen [1,2,3], Minh Doan [1], James F. Eliason [4], János Vincze [5], Lajos Szente [6], Ferenc Fenyvesi [7], Katalin Goda [1], Miklós Vecsernyés [7], Gábor Szabó [1] and Zsolt Bacso [1,2,7,*]

[1] Department of Biophysics and Cell Biology, Faculty of Medicine, University of Debrecen, 4032 Debrecen, Hungary; gutaynetzs@gmail.com (Z.G.-T.); gabgellen@staff.elte.hu (G.G.); vivavn@gmail.com (Q.-M.D.-X.); goda@med.unideb.hu (K.G.); szabog@med.unideb.hu (G.S.)
[2] Doctoral School of Molecular Cell and Immune Biology, University of Debrecen, 4032 Debrecen, Hungary
[3] MTA-ELTE Lendület Ion Mobility Mass Spectrometry Research Group, Department of Analytical
Chemistry, Institute of Chemistry, ELTE Eötvös Loránd University, 1053 Budapest, Hungary
[4] Great Lakes Stem Cell Innovation Center, Detroit, MI 48202, USA; jeliason@gmail.com
[5] Department of Physiology, Faculty of Medicine, University of Debrecen, 4032 Debrecen, Hungary;
vincze.janos@med.unideb.hu
[6] CycloLab Cyclodextrin Research & Development Laboratory, Ltd., 1097 Budapest, Hungary; szente@cyclolab.hu
[7] Department of Pharmaceutical Technology, Faculty of Pharmacy, University of Debrecen, 4032 Debrecen, Hungary; fenyvesi.ferenc@pharm.unideb.hu (F.F.); vecsernyes.miklos@pharm.unideb.hu (M.V.)
* Correspondence: bacso@med.unideb.hu

Running title: Membrane repair carries P-glycoprotein conformer

KEYWORDS: ABCB1 transporter, cyclodextrin, membrane repair, raft, trafficking, UIC2 antibody

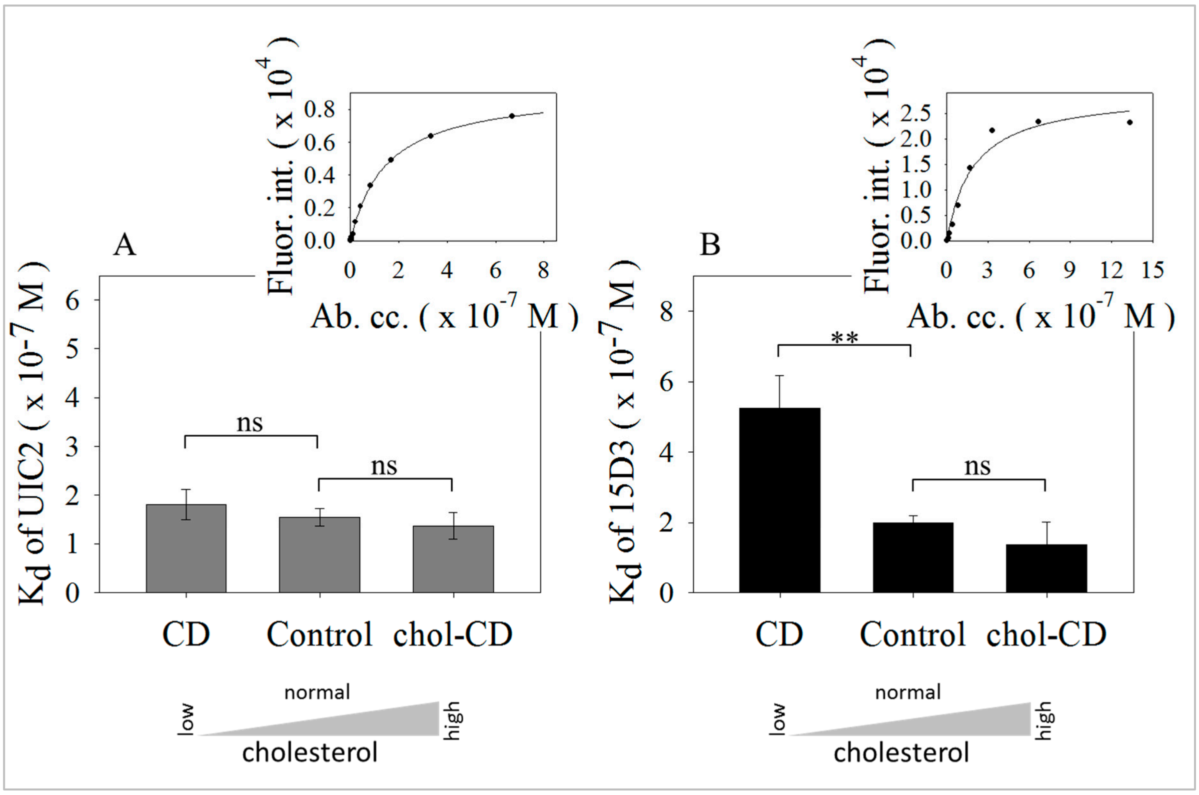

Figure S1 **The affinity of P-glycoprotein mAbs after cholesterol level modifications.** The affinity of the UIC2 mAb for P-glycoprotein did not change considerably after cholesterol level modifications of the cell membrane. Dissociation constant (K_d) values were calculated from saturation curves (inserts) for UIC2 and 15D3 mAbs. A: Affinity of the UIC2 mAb (ns: non-significant). B: Affinity of the 15D3 mAb (** p=0.0021 by unpaired, one-tail t-test.). Bars represent mean ± SD values of three independent experiments. Inserts show representative saturation curves of the control samples.

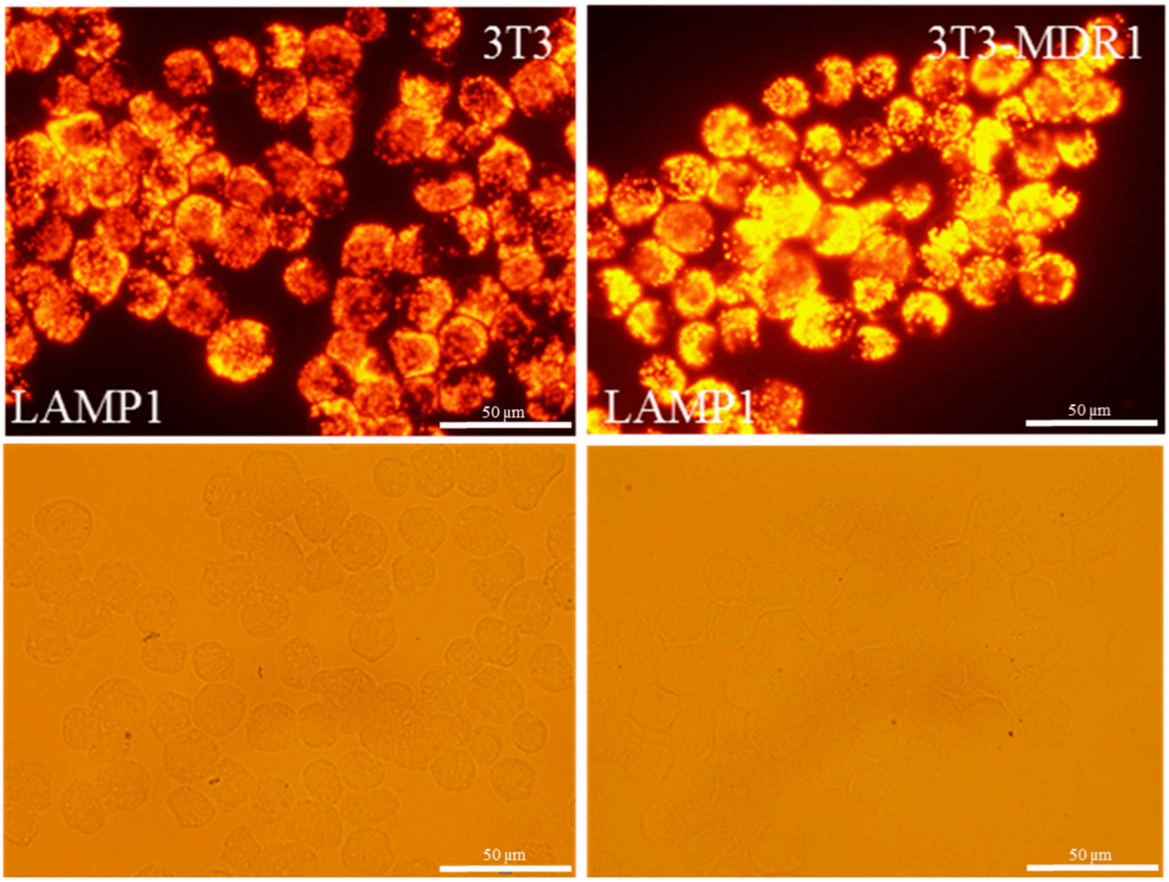

Figure S2 **The lysosomal LAMP1 protein expression in parental NIH-3T3 and 3T3-MDR1 cells.** Photomicrographs of NIH-3T3 and 3T3-MDR1 cells are shown after indirect immunofluorescence labeling. The scale bar represents 50 micrometers.

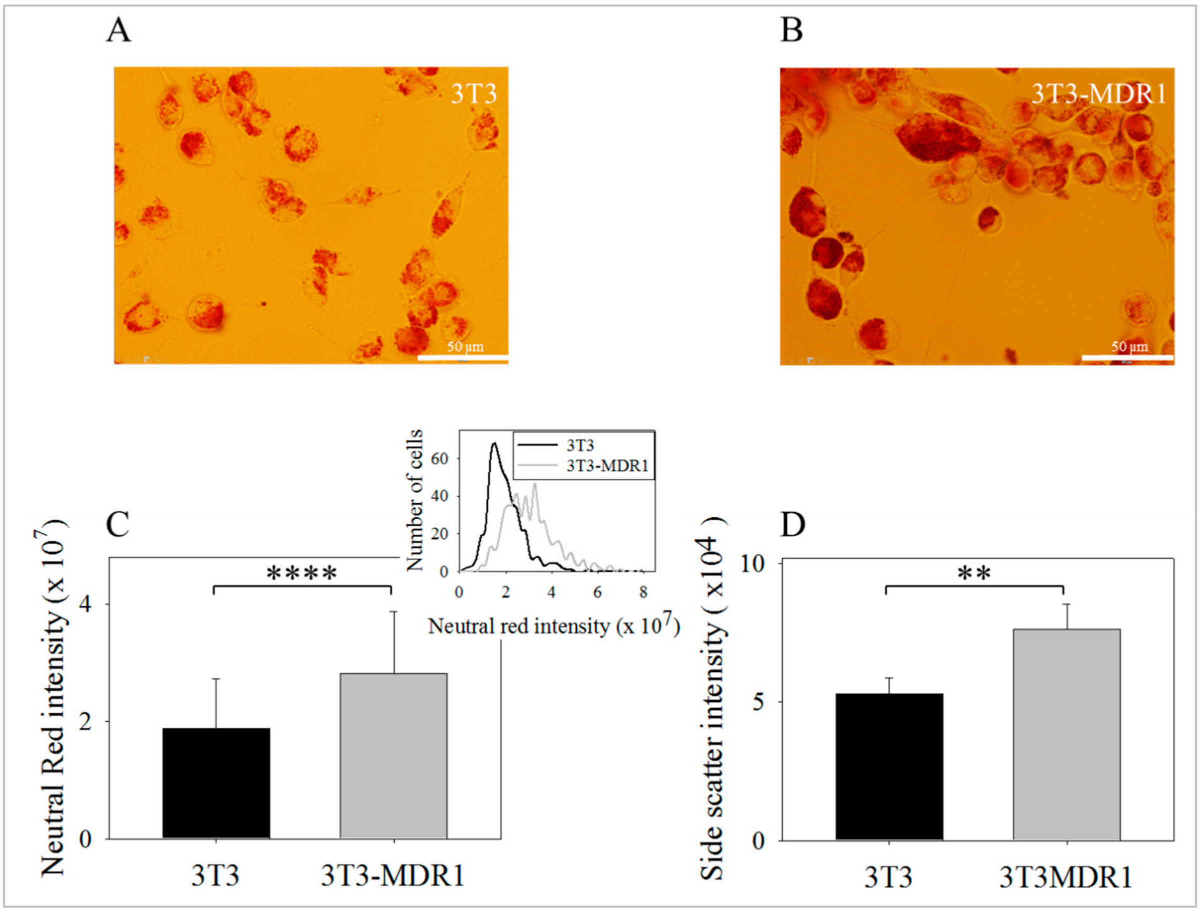

Figure S3 **As measured using quantitative imaging cytometry, 3T3-MDR1 cells contained more lysosomes than parental NIH-3T3 cells.** Microscopic images show NIH-3T3 (panel A) and 3T3-MDR1 (panel B) cells stained by the neutral red (NR) lysosomotropic chromatic dye. Scale bars in A and B are 50 micrometers. C: 3T3-MDR1 cells accumulated 1.5 times more NR in lysosomes than NIH-3T3 cells determined by laser-scanning cytometry. The median ± SD of NR absorbance was determined in approximately 500-700 cells of samples. One representative data set of five measurements. Insert shows the distribution of lysosomal content of NR in NIH-3T3 and 3T3-MDR1 cells (**** $p<0.0001$ by unpaired, one-tail t-test.) D: Light side scatter intensities of 3T3-MDR1 cells was 1.5 times higher than in NIH-3T3 cells measured by flow cytometry. Mean±SD, n=4. **$p=0.0026$ by unpaired one-tail t-test.

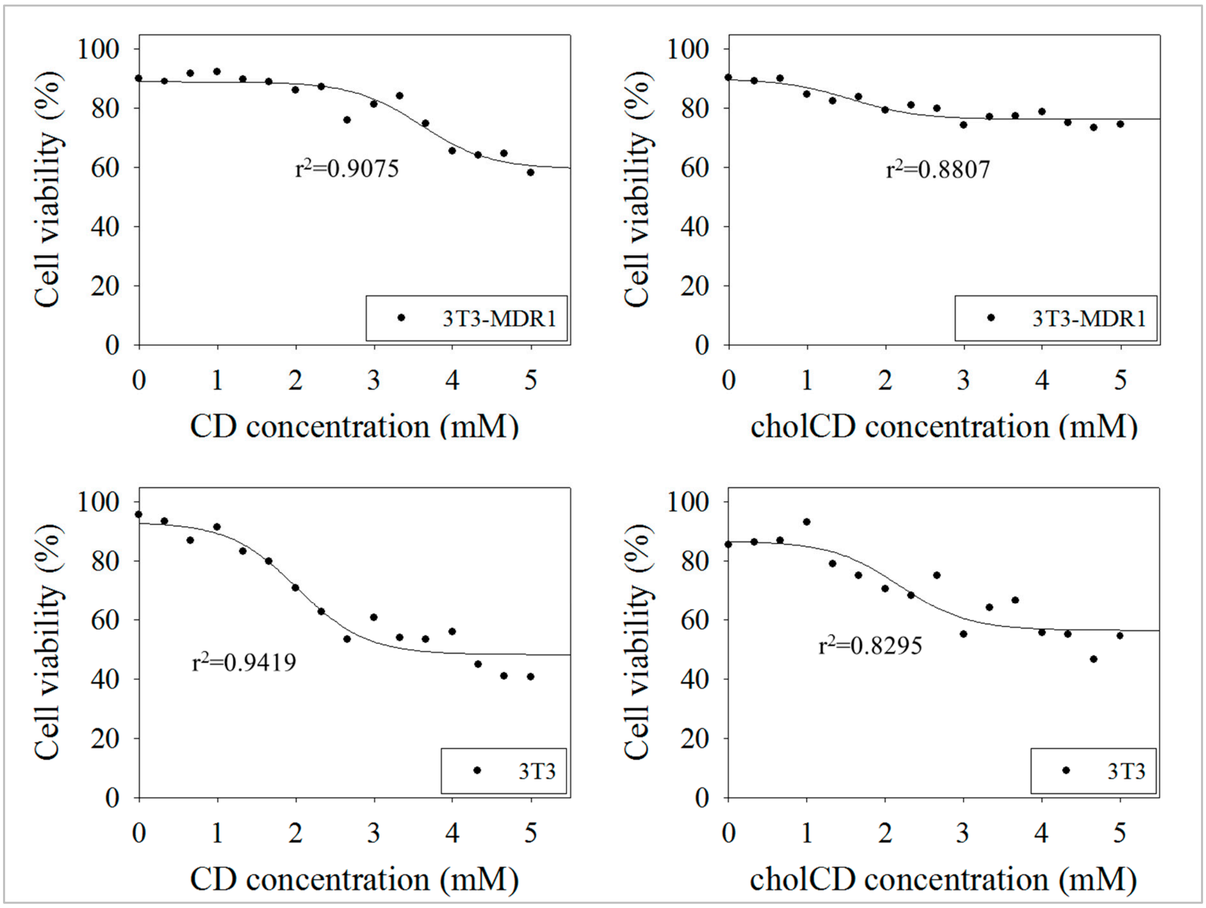

Figure S4 **Curve fitting of raw data of cell viability.** The curve fitting of a representative set of cell viability data in NIH-3T3 and 3T3-MDR1 cells after cholesterol modulations was done by Sigma Plot software according to materials and methods.

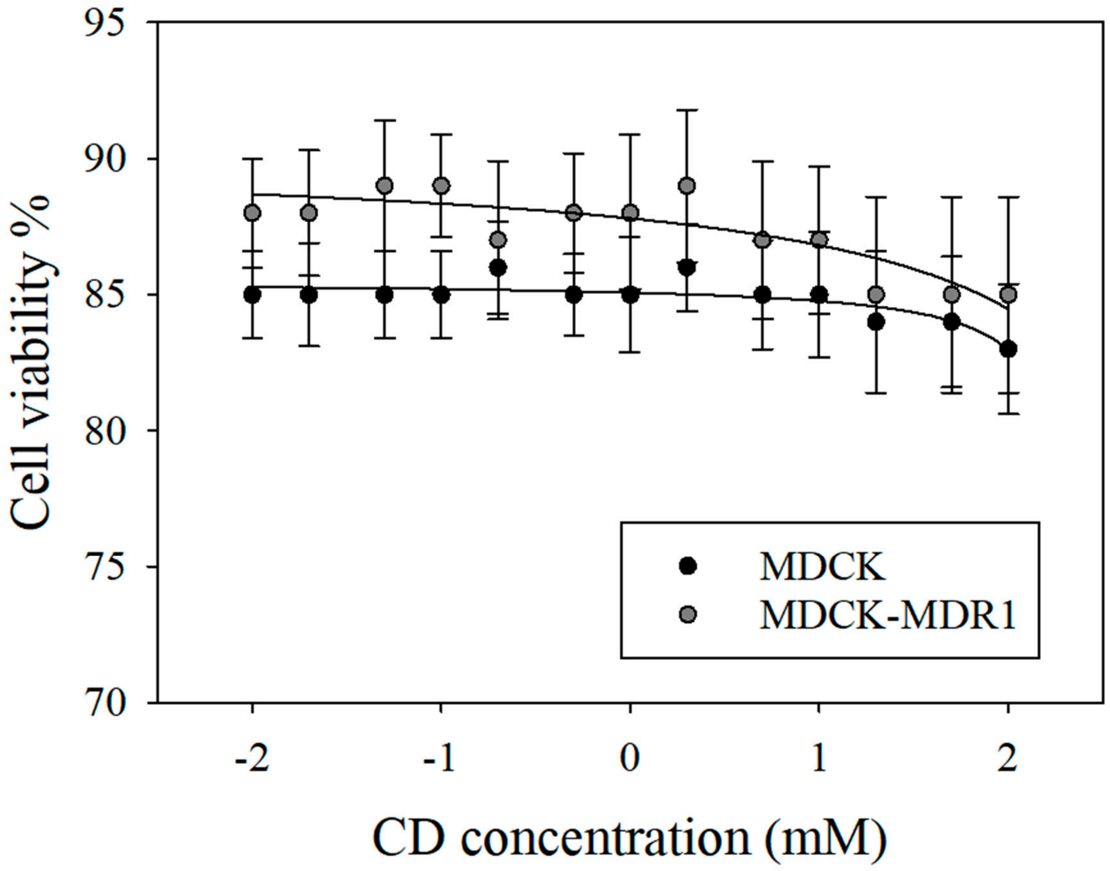

Figure S5. **Cell viability changes in multidrug-resistant MDCK-MDR1 and parental MDCK cells in case of whole-cell cholesterol changes.** MDCK cells and their multidrug-resistant human P-gp transfected pair were treated with increasing amounts of CD and chol-CD for cholesterol depletion and supplementation, respectively. The overall sensitivity of the parental MDCK cells for cholesterol perturbations was significantly larger than its multidrug-resistant human P-gp transfected pair since MDCK cell viability was significantly lower. Collected data points of the MDCK and MDCK-MDR1 cells were compared statistically from six independent experiments (three for cholesterol depletion (CD treatments) and three for cholesterol supplementation (chol-CD treatments)) in the range indicated on the graph of the different concentration cyclodextrin treatments (p=9.09×10-5 Student`s t-test).

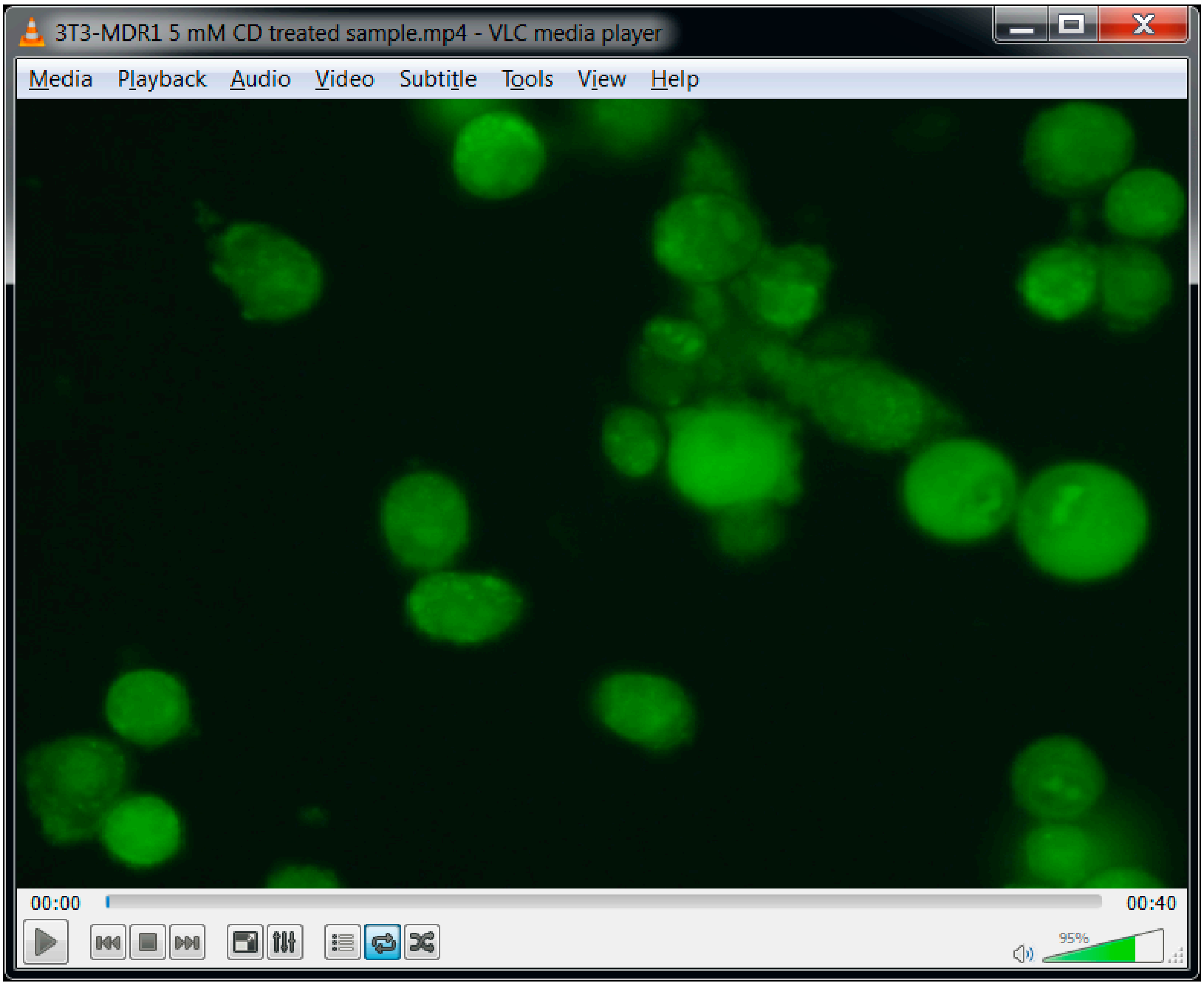

Video S1: 3T3-MDR1 5 mM CD treated sample. The effect of 5 mM CD treatment on lysosome membrane stability was investigated. A 20-minute CD treatment was followed by acridine orange staining and AO-loaded cells were continuously exposed to blue light irradiation in an epifluorescence microscope applying a 40x objective, and a video was recorded (details in Materials and Methods). Events of lysosomal ruptures correspond to noticeable fluorescence flashes in the video. Lysosomes had already started to burst at the first second of blue light illumination. Intensive loss of lysosomes could be observed in the first 20 seconds. A slight decrease in the number of lysosomes was detectable in these 5 mM CD-treated samples compared to the 3 mM CD-treated ones. However, the illumination time required to destroy all of the lysosomes did not decrease further.

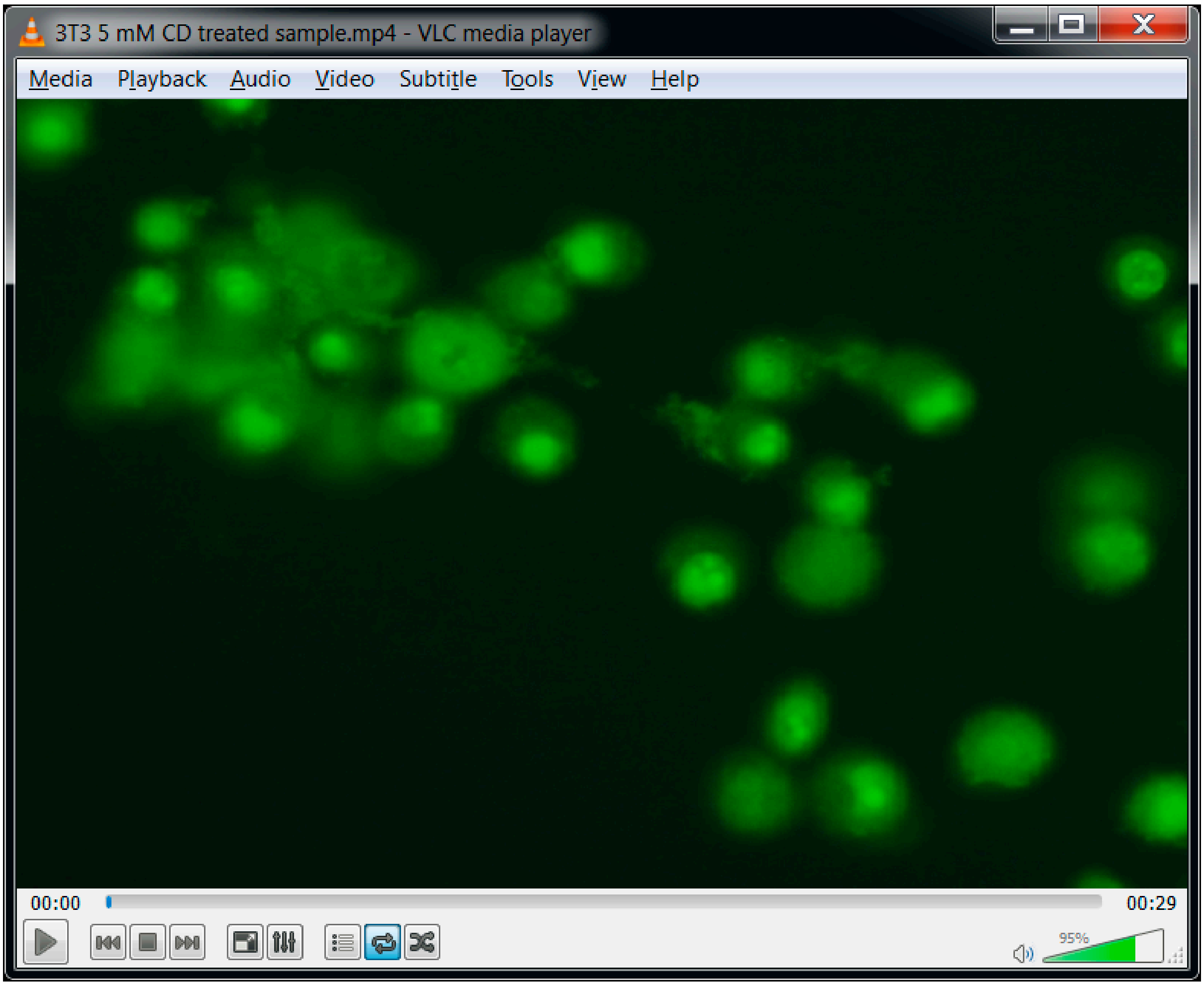

Video S2: NIH-3T3 5 mM CD treated sample. The identical 5 mM CD treatment, as in Video S1, eliminated NIH-3T3 cells' lysosomes before the AO-sensitized, blue light illumination-induced lysosomal membrane rupture. The 3 mM CD treatment significantly decreased the number of intact lysosomes before the illumination (video not shown), while the 5 mM CD treatment destroyed them entirely. Only the bleaching of nucleus staining is observable in this Video S2.
